# Assessing Caregiver Burden, Tasks, and Heart Rate Using Wearable Sensors: A Longitudinal Study of Informal Caregivers of Persons With Dementia and Older Adults

**DOI:** 10.7759/cureus.78059

**Published:** 2025-01-27

**Authors:** Kohei Kajiwara, Kimie Harada, Junko Shiraki, Tetsuo Ono, Takayo Nagata, Ayumi Morioka, Ayumi Ide, Maki Yoshimura, Ayako Ogata, Hiroko Noto, Jun Kako

**Affiliations:** 1 Faculty of Nursing, Japanese Red Cross Kyushu International College of Nursing, Munakata, JPN; 2 Department of Nursing, Imazu Red Cross Hospital, Fukuoka, JPN; 3 Department of Nursing, Imazu Red Cross Home-Visit Nursing Station, Fukuoka, JPN; 4 Department of Nursing, Imazu Red Cross Care Plan Center, Fukuoka, JPN; 5 Department of Care Plan Service, Care Plan Service Higashifukuma, Fukutsu, JPN; 6 Department of Health Science, Graduate School of Medical Sciences, Kyushu University, Fukuoka, JPN; 7 Graduate School of Medicine, Mie University, Tsu, JPN

**Keywords:** burden, dementia, heart rate, informal caregivers, technologies, wearable sensor

## Abstract

Purpose

In recent years, research on caregivers has highlighted the importance of integrating advanced technologies, such as wearable devices. Furthermore, when investigating the characteristics of persons with dementia (PWD), comparative analyses should be conducted based on the presence or absence of the condition. We aimed to elucidate the relationship between caregivers' subjective burdens, tasks, and heart rate (HR) using wearable sensors to objectively assess the health status of caregivers of PWD and older adults requiring long-term care.

Methods

The study recruited 21 caregivers of PWDs and older adults requiring long-term care between September 2022 and December 2024 from one hospital, two care plan centers, and visiting nursing stations in Fukuoka Prefecture, Japan. We collected the participants' sociodemographic data using a questionnaire survey for caregivers. We also measured the caregiver's HR, walking steps, and total sleep time using a wearable sensor.

Results

Data from 17 participants with no missing values were included in this analysis among the 21 caregivers who provided consent to participate in this study. The demographic variables of the caregivers and care recipients revealed that the caregivers were predominantly female (typical spouses). Most PWDs were diagnosed with atopic disease. No significant correlations were found between the short Japanese version of the Zarit Burden Interview (J-ZBI_8) and the following measures: caregiving years, activities of daily living (ADL), the 14-item Short-Memory Questionnaire (SMQ), caregiving time, the Caregiving Gratification Scale (CGS), total caregiving tasks, sleep time, walking steps, or conversation time. Caregiver burden was significantly associated with caregiving experience and continued caregiving. We observed no significant differences in the average HR for pre- and post-caregiving tasks. Significant differences were found in the maximum pre- and post-caregiving HR. While caregiver burden showed a high-scoring trend and positive perception showed a low-scoring trend, none of the variables differed according to the presence or absence of PWDs.

Conclusions

These results indicate that subjective appraisal of caregiver burden was not significantly associated with HR change during caregiving tasks. No differences were observed in the behaviors of caregivers with or without dementia. It is necessary to measure subjective and objective appraisals using wearable sensors to better understand caregivers' situations.

## Introduction

The number of persons with dementia (PWD) is projected to increase from 55 million in 2019 to 139 million by 2050 [1]. Most studies on family caregivers of persons with dependency have underlined the importance of managing the caregiver burden, which can lead to fatigue and a decline in mental health [2,3]. In particular, caregivers of PWD experience difficulty managing the behavioral and psychological symptoms of dementia (BPSD), which are strongly related to a greater caregiver burden [4,5].

Previous studies have highlighted the importance of both the positive aspects of caregiving and the caregiver burden [6,7]. Numerous intervention studies using subjective burden assessments have reported positive outcomes, emphasizing the effectiveness of educational interventions and comprehensive caregiver support as primary focal points. Many intervention studies using the subjective burden have reported that interventions are effective as educational interventions and that comprehensive support for caregivers is the primary focus [8-11]. Furthermore, the efficacy of online interventions in enhancing the positive aspects of caregiving among caregivers of PWD was highlighted. The online facilitators provided participants with emotion regulation skills aimed at fostering positive emotions, resulting in a demonstrated increase in the positive dimensions of caregiving. In addition, the effectiveness of online interventions has been suggested [12].

Recent studies have focused on the subjective caregiving appraisal of caregivers as well as actual caregiving-related tasks, exercise, sleep, health-related behaviors, and physical health, with both objective data and caregiver burden being considered [13-16]. Kajiwara et al. demonstrated an association between caregiver burden and blood pressure [15]. Other studies have determined that reduced total objective sleep time is significantly related to caregiver burden among family caregivers [16]. Moreover, research on employing technology to evaluate caregiver burden has recently gained increasing attention [17]. Wearable sensor technologies have emerged as a central focus, and active investigations are being pursued across diverse disciplines [18,19]. However, a study on caregivers using wearable sensors noted that their effectiveness remained uncertain [19]. Furthermore, a systematic review of previous studies revealed scant research on the use of technology [17]. In addition, to examine the characteristics of PWD, it is important to make comparisons according to the presence or absence of dementia.

Therefore, this study aimed to elucidate the relationship between caregivers' subjective burdens, caregiving tasks, and heart rate (HR), measured using wearable sensors, to objectively assess the health status of caregivers of PWD and older adults requiring long-term care.

## Materials and methods

Participants and study design

In this study, we used a self-administered questionnaire survey and a longitudinal survey measuring caregiving task behaviors over a 72-hour period. The participant sample size was set at 20 or more based on sample sizes utilized in previous studies [15,16]. We recruited 21 caregivers of PWDs between September 2022 and December 2024 from a hospital, two care plan centers, and visiting nursing stations in Fukuoka Prefecture, Japan. To meet the inclusion criteria, participants had to (1) be family caregivers of PWDs or older adults in need of long-term care, (2) provide care for more than five hours/week at home, and (3) be aged >18 years. The exclusion criteria for the participants were defined as follows: (1) cases where the attending physician or facility administrator clinically determined that PWDs or older adults requiring long-term care were suffering from a life-threatening severe illness or were in the terminal stage; (2) cases where the attending physician or facility administrator determined that the caregiver had a life-threatening severe illness; and (3) cases where other circumstances, as assessed by the responsible personnel at the collaborating research facility, were deemed to render the execution of the study infeasible.

Using a questionnaire designed for caregivers, we collected data on the sociodemographic characteristics of caregivers and PWDs. Caregiver HR, walking steps, conversation time, and total sleep time were measured using a wearable sensor (SilmeeTM W22, TDK Corporation, Tokyo, Japan) [20]. The sensor was worn on the wrist for three consecutive 24-hour periods (i.e., 72 hours). The caregiving tasks during the 72-hour period were investigated using a self-administered recording form.

This study was approved by the Ethics Committee of the Japanese Red Cross Kyushu International College of Nursing (approval no. 22-017). All participants provided written informed consent.

Outcome measures

The general characteristics of the caregivers included age, sex, relationship with the PWD, number of years of caregiving, and hours per day spent caring. The general characteristics of PWDs included sex, dementia diagnosis, use of services for older adults, and age. Activities of daily living (ADL) were assessed using a four-point Likert scale developed by the Ministry of Health, Labor, and Welfare to assess the abilities of disabled older adults and determine their degree of independence in daily living.

Referring to previous studies [21], the change and negative impact of COVID-19 on the content of care was measured using a five-point Likert scale.

The wearable sensors measured various lifestyle factors [20], including HR, number of steps, conversation time, and total sleep time. HR, walking steps, and sleep time were measured at one-minute intervals. During this time, the sensor was removed only for certain activities, such as showering and bathing, if it interfered with the caregiver's activities.

Cognitive impairment in care recipients was assessed using the 14-item Short-Memory Questionnaire (SMQ) [22,23]. The SMQ was developed to evaluate the degree of cognitive impairment in the daily life of care recipients. The total score ranged from 4 to 46, with a score of <40 indicating likely dementia.

Caregiver burden was assessed using the short Japanese version of the Zarit Burden Interview (J-ZBI_8) [24,25]. The J-ZBI_8 comprises eight items in two subscales: personal strain (five items) and role strain (three items), based on the factor structure of the Zarit Burden Interview [21]. Each item was rated on a five-point Likert scale, with total scores ranging from 0 to 32, where higher scores reflect greater caregiver burden.

The Caregiving Gratification Scale (CGS) was used to measure caregivers' positive appraisal of their caregiving [26]. The scale comprises eight items, each rated on a four-point Likert scale, with total possible scores ranging from 0 to 24 points. The reliability and validity of the CGS have been verified in Japan [26,27]. Cronbach's alpha coefficients were observed to range between 0.788 and 0.828 [26].

Upon completing the anonymous caregiving task record questionnaire (assistance with meals, toileting, walking, bathing, and clothes changing) [15], the participants wore the wearable sensors at home for 72 hours.

With reference to previous research [27], the participants' desire to continue caregiving at home over the next two years was assessed as a subjective feeling on a five-point Likert scale.

Data analysis

We calculated the mean 10-minute HR during pre- and post-caregiving tasks based on the subject's self-administered care behavior record. The post-maximum rate was also used to examine changes in caregivers' HR in response to caregiving tasks [15]. We computed the percentage change in each caregiver's HR and selected the median percentage change as an independent variable. Variable and correlation analyses were conducted using Spearman's rank correlation coefficients to investigate any associations between caregivers' subjective appraisals and the change rate in caregivers' HR. The mean maximum HR rate was calculated both before and after the caregiving tasks, as well as the average maximum HR rate recorded within the 10-minute period following the completion of these tasks. The dependent-sample t-test was performed to determine significant differences in HR between pre- and post-caregiving tasks and the maximum number of post-caregiving tasks. The Mann-Whitney U test was used to examine differences in caregivers with and without dementia. Statistical analyses were performed using IBM SPSS Statistics for Windows, Version 29 (Released 2023; IBM Corp., Armonk, New York), and significance was set at p < 0.05.

## Results

Participant characteristics

Data from 17 participants with no missing values were included in this analysis among the 21 caregivers who provided consent to participate in this study. The demographic variables of the caregivers are summarized in Table 1, highlighting that the majority are female (typically spouses). For the PWD, most of them were diagnosed with atopic disease. Table 2 presents the demographic characteristics of the care recipients.

**Table 1:** Sociodemographic characteristics of the caregivers PWD: persons with dementia; CGS: Caregiving Gratification Scale; HR: heart rate; SD: standard deviation

Variables	n (%)	Mean	SD
Sex			
Men	2 (11.8)	-	-
Women	15 (88.2)	-	-
Relationship to the PWD			
Spouse	9 (52.9)	-	-
Daughter	6 (35.3)	-	-
Daughter-in-law	2 (11.8)	-	-
Age (range: 54–83 years)	-	68.1	7.4
Caregiving years (range: 0.2–20)	-	5.8	5.5
Caregiving hours per day (range: 2–24)	-	13.4	7.3
Total number of caregiving tasks (72 hours)	-	17.2	9.4
J-ZBI_8	-	12.5	8.3
CGS	-	17.1	5.6
Changes in care provided by COVID-19 (range: 1–5)	-	2.4	1.5
Negative effects due to COVID-19 (range: 1–5)	-	2.4	1.3
72-hour HR	-	66.6	6.5
HR (pre-caregiving)	-	66.6	6.4
HR (post-caregiving)	-	67.2	7.5
HR (maximum value at post-caregiving)	-	83.5	10.8
72-hour walking total steps	-	13,690.5	8,801.2
72-hour total sleep time (min)	-	1,585.8	413.3
Conversation time (minutes)	-	724.1	355.2
HR anteroposterior change (%)	-	100.4	3.5
HR maximum rate of change (%)	-	119.2	5.6

**Table 2:** Sociodemographic characteristics of the care recipients SMQ: Short-Memory Questionnaire; SD: standard deviation

Variables	n (%)	Mean	SD
Sex			
Men	12 (70.6)	-	-
Women	5 (29.4)	-	-
Diagnosis			
Alzheimer’s	8 (47.1)	-	-
Vascular	1 (5.9)	-	-
None (not clear)	7 (41.2)	-	-
Older adult services			
Day-care service	11 (64.7)	-	-
Day rehabilitation	5 (29.4)	-	-
Visiting nursing care	8 (47.1)	-	-
Visiting rehabilitation	6 (35.3)	-	-
House call medical services	6 (35.3)	-	-
Short-stay service	2 (11.8)	-	-
Visiting older adults care	2 (11.8)	-	-
Other	5 (29.4)	-	-
Age (range: 68–97 years)	-	82.8	8.4
Activities of daily living score (range: 1–4)	-	2.1	0.8
SMQ	-	11.0	10.8

Relationship between caregiver burden and variables

Spearman's rank correlation coefficients are presented in Table 3. No significant correlations were found between the J-ZBI_8 and the caregiver years, ADL, SMQ, caregiving time, CGS, total caregiving tasks, sleep time, walking steps, or conversation time. However, caregiver burden was significantly associated with caregiving experience and the continuation of caregiving.

**Table 3:** Spearman's rank correlation coefficients between J-ZBI_8 and caregiver and care recipient characteristics ADL: activities of daily living; HR: heart rate; SMQ: Short-Memory Questionnaire; CGS: Caregiving Gratification Scale

Baseline (J-ZBI_8)	n	ρ	p
Caregiving years	17	0.523	0.031
ADL	17	0.338	0.185
SMQ	17	–0.246	0.340
Caregiving time	17	0.191	0.463
CGS	17	–0.099	0.706
Continuation of caregiving	17	–0.701	0.002
Total caregiver tasks	17	0.324	0.204
Sleep time	17	0.169	0.516
Walking steps	17	–0.045	0.863
Conversation time	16	–0.416	0.109
HR anteroposterior change (%)	17	–0.205	0.430
HR maximum rate of change (%)	17	0.282	0.273

Changes in HR during caregiving tasks

The t-test results indicated differences in the HRs for pre- and post-caregiving and post-caregiving maximum rates. We observed no significant differences in the average HR for pre- and post-caregiving tasks (t = -0.688, p = 0.501). Significant differences were found in the pre- and post-caregiving maximum HR (t = -9.587, p < 0.001).

Comparison by presence of dementia

The Mann-Whitney U test results are presented in Table 4. Caregivers' burden showed a high-scoring trend, while positive perception showed a low-scoring trend. None of the variables differed according to dementia status.

**Table 4:** Mann–Whitney U test results for comparisons with and without a diagnosis of dementia CGS: Caregiving Gratification Scale; HR: heart rate

Variables	Dementia	No Dementia	p
J-ZBI_8	14.9 ± 10.4	9.5 ± 5.3	0.277
CGS	15.1 ± 6.4	19.9 ± 3.4	0.059
Continuation of caregiving	3.9 ± 1.3	4.5 ± 0.8	0.370
Total caregiver tasks	15.9 ± 6.5	20.0 ± 11.4	0.277
Sleep time	1668.1 ± 507.3	1461.1 ± 301.9	0.321
Conversation time	640.5 ± 351.0	807.6 ± 362.3	0.328
HR anteroposterior change (%)	101.6	99.2	0.277
HR maximum rate of change (%)	119.1	118.6	0.963

## Discussion

Our study revealed that caregiver burden, as a subjective appraisal, was not significantly associated with HR changes in caregiving tasks. In addition, changes in pulse rate variability with and without dementia were not significantly different; however, we did observe significant HR changes in the pre- and post-caregiving maximum rates.

In this study, we did not find an association between subjective caregiver burden, HR anteroposterior change, or HR maximum rate of change. The results of this study are similar to those of a previous study that indicated no correlation between subjective caregiver burden and changes in the pre- and post-caregiving HR of caregivers, as well as the maximum change in HR after a caregiving task [15]. Another study assessed the potential correlation between objective measurements obtained using wearable sensors and subjective outcome measures related to the health and well-being of caregivers and PWD, including depression, anxiety, quality of life, and burden of care [18]; no significant correlations were found between objective sleep measures and depression. However, Ryuno et al. [16] indicated that total sleep time measured using the ActiGraph was significantly associated with care burden among care receivers receiving long-term care at home [16]. Our results suggest a transient increase in HR due to caregiving. HR variability has also been employed as an outcome measure in heart-focused breathing interventions targeting caregivers; however, no statistically significant effects have been demonstrated [28]. Although no previous studies have produced consistent findings, current evidence indicates that subjective and objective data do not always align, underscoring the importance of comprehensively assessing caregivers from multiple perspectives. Therefore, we believe it is necessary to measure subjective and objective appraisals using wearable sensors to better understand caregivers' situations.

In this study, no significant differences were observed in the changes in caregiving behaviors of those with or without dementia. BPSD has been shown to significantly influence caregiver burden [29]. Another study showed that dementia was the only characteristic associated with caregiver burden [30]. The analysis of caregiving tasks and HR patterns in this study revealed that differences may not be discernible based on the presence or absence of dementia. However, objective data may present challenges in effectively capturing the distinctions associated with dementia. A previous study highlighted that the potential applications of wearable sensors remain incompletely explored, with large gaps in our understanding due to the limitations in the available evidence among PWD caregivers. However, the practical implementation of technological devices has demonstrated wide applicability within the field of nursing care. Digitally based dementia education can have a positive effect on caregiver depression and the ability to manage behavioral issues in caregiving, which could improve the caregiving experience [31]. Wearable sensors can be used with minimal disruption to the routines of busy caregivers, making them valuable tools for objectively visualizing the caregiving burden experienced by those caring for PWDs in community settings.

This study has some limitations. A primary limitation of this study is its small sample size; however, prior research has demonstrated that most studies employing similar technologies often involve sample sizes of fewer than 20 participants [32]. Second, given the measurement of daily life data using wearable sensors, numerous confounding factors may have influenced the pulse readings. Third, the study included PWDs and older adults requiring care; however, even those without a formal dementia diagnosis were classified as cognitively impaired based on the SMQ. The inclusion criteria for patients without dementia who require long-term care warrant further consideration in future studies. Fourth, measurements using wearable sensors are conducted in home settings, occasionally necessitating temporary removal of the device during bathing, which may disrupt continuous data collection. Fifth, because measurements are scheduled based on caregivers' availability for participation, the 72-hour monitoring period may vary depending on individual caregiving circumstances. Despite these limitations, care providers should consider objective HR measurement using wearable sensors. Future studies should focus on interventional research using wearable sensors to obtain objective data.

## Conclusions

These results indicate that subjective appraisal of caregiver burden was not significantly associated with HR change rate during caregiving tasks. No significant differences were observed in the changes in the behaviors of caregivers of patients with or without dementia; however, caregiving tasks induced a transient increase in HR. These findings suggest that HR variability can serve as an objective indicator of caregiver burden, thus complementing subjective assessments. We consider it essential to utilize wearable sensors to assess both subjective and objective evaluations for a deeper understanding of caregivers' circumstances.
